# Chromosomal Translocations in Black Flies (Diptera: Simuliidae)—Facilitators of Adaptive Radiation?

**DOI:** 10.1371/journal.pone.0158272

**Published:** 2016-06-27

**Authors:** Peter H. Adler, Oyunchuluun Yadamsuren, William S. Procunier

**Affiliations:** 1 Department of Agricultural and Environmental Sciences, Clemson University, Clemson, South Carolina, United States of America; 2 Department of Biology, Mongolian National University of Education, Ulaanbaatar, Mongolia; 3 Department of Psychology, Nipissing University, North Bay, Ontario, Canada; Virginia Tech, UNITED STATES

## Abstract

A macrogenomic investigation of a Holarctic clade of black flies—the *Simulium cholodkovskii* lineage—provided a platform to explore the implications of a unique, synapomorphic whole-arm interchange in the evolution of black flies. Nearly 60 structural rearrangements were discovered in the polytene complement of the lineage, including 15 common to all 138 analyzed individuals, relative to the central sequence for the entire subgenus *Simulium*. Three species were represented, of which two Palearctic entities (*Simulium cholodkovskii* and *S*. *decimatum*) were sympatric; an absence of hybrids confirmed their reproductive isolation. A third (Nearctic) entity had nonhomologous sex chromosomes, relative to the other species, and is considered a separate species, for which the name *Simulium nigricoxum* is revalidated. A cytophylogeny is inferred and indicates that the two Palearctic taxa are sister species and these, in turn, are the sister group of the Nearctic species. The rise of the *S*. *cholodkovskii* lineage encompassed complex chromosomal and genomic restructuring phenomena associated with speciation in black flies, *viz*. expression of one and the same rearrangement as polymorphic, fixed, or sex linked in different species; taxon-specific differentiation of sex chromosomes; and reciprocal translocation of chromosome arms. The translocation is hypothesized to have occurred early in male spermatogonia, with the translocated chromosomal complement being transmitted to the X- and Y-bearing sperm during spermatogenesis, resulting in alternate disjunction of viable F1 translocation heterozygotes and the eventual formation of more viable and selectable F2 translocation homozygous progeny. Of 11 or 12 independently derived whole-arm interchanges known in the family Simuliidae, at least six are associated with subsequent speciation events, suggesting a facilitating role of translocations in adaptive radiations. The findings are discussed in the context of potential structural and functional interactions for future genomic research.

## Introduction

The role of translocations in genomic and karyotypic evolution of eukaryotes is apparent—from their involvement in speciation processes and their use as tools in experimental genetic breeding systems [1,2] to their causal molecular associations in human cancerous and noncancerous diseases [3,4]. Examples also come from classic cytological work with plants, such as the evening primrose *Oenothera*. Members of *Oenothera* exhibit permanent translocation heterozygosity, conferred by meiotic ring formation, and in the extreme, can comprise the full karyotype (*2n* = 14) of all seven bivalents [5]. Other examples include the adaptive radiation of mammalian species, such as mice, through Robertsonian fusions and whole-arm interchanges [6] and the discovery that complex structural rearrangements can occur in certain cancers defined as chromothripsis [7]. These processes include not only translocations, but also duplications (copy-number changes), DNA-repair modifications, expression-profiling differences, and effects on pathway regulation for genes with various three-dimensional spatial chromatin reorganization and architecture [8–10].

In insects of the family Simuliidae (black flies), restructuring of the chromosomal complement (typically *2n* = 6) is associated with evolution and speciation [11–13]. The vast majority of species in the family are defined by unique rearrangements, of which the most common are paracentric inversions, followed by band enhancements or heterobands [14]. Their discovery is enabled by the detailed banding patterns of the giant, polytene chromosomes in the larval silk glands [12,15] and the adult Malpighian tubules [16,17].

Major chromosomal translocation types exist in black flies. A special situation, for instance, has occurred in some Norwegian populations of the dichromosomic (*n* = 2) *Cnephia pallipes* (Fries) (formerly *C*. *lapponica*). Males are whole-arm interchange heterozygotes; functionally, they behave as *n* = 1 [18]. Females have the normal chromosome I of related species, plus a new longer chromosome, designated chromosome II, that has resulted from fusion of transposed centromeres to the telomeric ends of the standard chromosomes II and III, producing the following sequential banding: IIIL + IIIS (fused centromere regions) + IIS + IIL. In the interchange heterozygotes, males exhibit Y-chromosome differentiation with Y_1_ = IS + Ce IIS + IIL and Y_2_ = Ce IIIS + IIIL, where Ce = the centric region containing the centromere. Fidelity of balanced chromosome types is maintained through alternate disjunction during meiosis.

Additional translocation examples in black flies are uncommon, whether mid-arm or whole-arm [19]. Of the 15 or so independently derived translocations in the Simuliidae, 80% involve whole-arm interchanges (including the type described in this study). Additional translocation types include the transfer of an entire arm to the terminus of its sister arm [20], relocation of a portion of one arm to a nonhomologous arm [21], and transfer of two adjacent bands to another arm [22]. The dearth of fixed, intra-arm translocations might be related to the detrimental consequences for heterozygotes, such as meiotic disjunction problems [21]. The ease with which one or a few translocated bands could be overlooked might, in part, explain their (apparent?) rarity.

Of the six possible whole-arm interchange combinations, one of the least represented is the IS + IIIL, IL + IIIS combination, known in *Simulium nigricoxum* Stone [23], a member of the *S*. *malyschevi* species group. This species group includes about 40 nominal species in the Northern Hemisphere where the immature stages inhabit swift water, and the female flies feed on the blood of mammalian hosts [20]. None of the other 12 chromosomally studied members of the *S*. *malyschevi* species group previously were known to carry a whole-arm interchange [12].

We present the discovery of a whole-arm interchange in two additional taxa related to *Simulium nigricoxum*, all three of which are members of the *S*. *cholodkovskii* lineage. All of these taxa have an interchange identical to that reported previously from a single population of *S*. *nigricoxum* Stone in Yukon, Canada [23]. *Simulium nigricoxum*, a name originally applied to Nearctic populations, was synonymized in 2004 with *S*. *decimatum* Dorogostaisky, Rubtsov & Vlasenko, although the possibility that cryptic species were involved was suggested [20]. We provide full resolution of the chromosomal banding patterns of these three taxa relative to the standard (central) banding sequence for the subgenus *Simulium*, interpret the taxonomic implications including a test of the species status of *S*. *nigricoxum* separate from *S*. *decimatum*, infer the phylogenetic relationships chromosomally, place the interchange discovery in the context of all known whole-arm interchanges in the Simuliidae, examine the association of whole-arm interchanges with adaptive radiations in the family, and comment on potential structural and functional interactions for future genomic research.

## Materials and Methods

### Ethics Statement

Collections of larvae in Mongolia were made on public lands; permits were not required. Specimens from the Northwest Territories were collected under a scientific research permit issued in 2002 to D. C. Currie (Royal Ontario Museum). No collections involved endangered or protected species.

### Collection and identification of material

Larvae and pupae were collected from stones and trailing vegetation at three sites in Canada and Mongolia (Table 1) and fixed in three changes of 1:3 acetic ethanol. Site 1 (17 m wide) was about 170 river km (ca. 112 straight km) downstream from Site 2 on Mongolia’s Tuul River. Site 2 (9 m wide) was about 160 river km (ca. 130 straight km) downstream from the site where *S*. *acrotrichum* Rubtsov previously was collected and analyzed chromosomally [24]. No larvae of the *S*. *cholodkovskii* lineage were collected at the site with *S*. *acrotrichum*, which receives pollution from Ulaanbaatar. Site 1 was influenced by livestock grazing and placer gold mining (suspended solids 136 mg/l, turbidity 106 NTU, conductivity 232 μS/cm), whereas Site 2 was affected by heavy livestock grazing without mining (suspended solids 213 mg/l, turbidity 167 NTU, conductivity 189 μS/cm). The river at both sites suffered from erosion and had basic pH (8.4–8.8). Site 3 (ca. 200 m wide) in Canada was about 7400 km from the nearest Mongolian site. The river at this site was acidic (pH 6.6).

**Table 1:** Collections of larvae of the *Simulium cholodkovskii* lineage used in chromosomal analyses.

Site No.	Location	Latitude, longitude	Elevation (m asl)	Date	Larvae (*n*)
1	Mongolia, Töv Aimag^1^, Zaamar, Tuul River	48°29'15"N 104°33'02"E	937	3 August 2013	90
2	Mongolia, Töv Aimag, Öndörshireet, Tuul River	47°31'40"N 105°00'40"E	1017	3 August 2013	26
3	Canada, Northwest Territories, Thelon River, Lookout Point	64°09’30”N 102°32’33”W	114	6 July 2002	22

^1^ Aimag = Province.

Larvae and associated pupae were identified as *S*. *cholodkovskii* and *S*. *decimatum* on the basis of morphological characters in keys and descriptions [20,25]. For the Canadian population identified as *S*. *decimatum*, we applied the name *S*. *nigricoxum*, which was originally (pre-2004) used for North American populations of *S*. *decimatum* [20], to highlight our test of its possible species status.

### Chromosomal mapping and analyses

Penultimate and early ultimate larval instars were cut transversely at abdominal segment IV, and the posterior portion was opened ventrally and stained using the Feulgen procedure [26]. The silk glands (with polytene nuclei) and one gonad (for linking gender to chromosomal rearrangements) of each larva were removed with fine needles, transferred to a drop of 50% acetic acid on a glass slide, and squashed, with thumb pressure, under a coverslip. High-quality chromosomes were photographed under oil immersion on an Olympus BX40 compound microscope. Adobe PhotoShop Elements 8 was used to construct chromosomal maps from scanned photographic negatives of chromosomes of larvae from Mongolia’s Tuul River (Sites 1 and 2), unless otherwise specified in the figure captions. Larval carcasses and photographic negatives of chromosomes were deposited in the Clemson University Arthropod Collection, Clemson, SC.

Selected chromosomal markers for the short (S) and long (L) arms of each of the three chromosomes (I, II, and III) are identified on our maps, following established terminology [27], to facilitate arm recognition. The centromere (C) of each chromosome is identified on our maps as a distinct band, and is distinguished from the centric region (Ce), which includes the centromere band and the immediate flanking area on either side. All chromosomal rearrangements were resolved with respect to the standard banding sequence for the subgenus *Simulium* by comparing the banding patterns of each larva against the subgeneric standard map [27,28]. The standard is the most central banding sequence from which all other band sequences in the subgenus can be resolved in the fewest number of steps. We compared the fine banding in the centromere region of our material not only with that of the subgeneric standard, but also with that of a related species, *S*. *malyschevi* Dorogostaisky, Rubtsov & Vlasenko, in the same species group. Section numbers (1–100) on our maps refer to those of the subgeneric standard.

Fixed inversions within a taxon are indicated by italics, whereas autosomal polymorphisms and sex-linked inversions are indicated by roman type. We used the same numbers for the same inversions (i.e., *IL-1*, *IIS-1*, *IIL-1*, *IIL-2*, *IIIS-1*, and *IIIL-2*) previously found in related species [24, 28]. Newly discovered inversions were numbered, typically in order of discovery, according to the next available number in each arm. Heterobands (Hb) were named for the sections in which they occurred (e.g., IIS 43Hb), as were deletions (d) of bands (i.e., 85d). Inversions are indicated on our maps by brackets or arrows—solid if fixed or polymorphic but dashed if linked to the X chromosome and dotted if linked to the Y. Any of the three chromosomes can serve as the sex chromosome. The X and Y can be microscopically indistinguishable (X_0_Y_0_) or one or the other can be partially or completely linked with a structural rearrangement, such as an inversion [19]. Common polymorphisms were tested for Hardy-Weinberg equilibrium.

### Phylogenetic inference

We inferred the phylogenetic relationships on the basis of uniquely shared, derived chromosomal rearrangements [24]. We first resolved all rearrangements relative to the *Simulium* subgeneric standard [27,28]. We then compared the IS, IIL, IIIS, and IIIL arms of the subgeneric standard and the breakpoints for inversions in the *S*. *cholodkovskii* lineage with the relevant sections of the standard sequences in two outgroups: *Simulium* (*Boophthora*) *erythrocephalum* De Geer and *Simulium* (*Psilozia*) *vittatum* Zetterstedt. IL could be resolved only with regard to *S*. *erythrocephalum*; IIS was too scrambled to resolve relative to either outgroup.

## Results

We analyzed all banding patterns of the 138 prepared larvae (72 females, 66 males). Body pigmentation of male larvae typically was brownish and that of females grayish, reflecting the sexual dimorphism described for larvae of other members of the *S*. *malyschevi* group, such as *S*. *acrotrichum* [24] and *S*. *defoliarti* Stone & Peterson [20].

**Fig 1:**
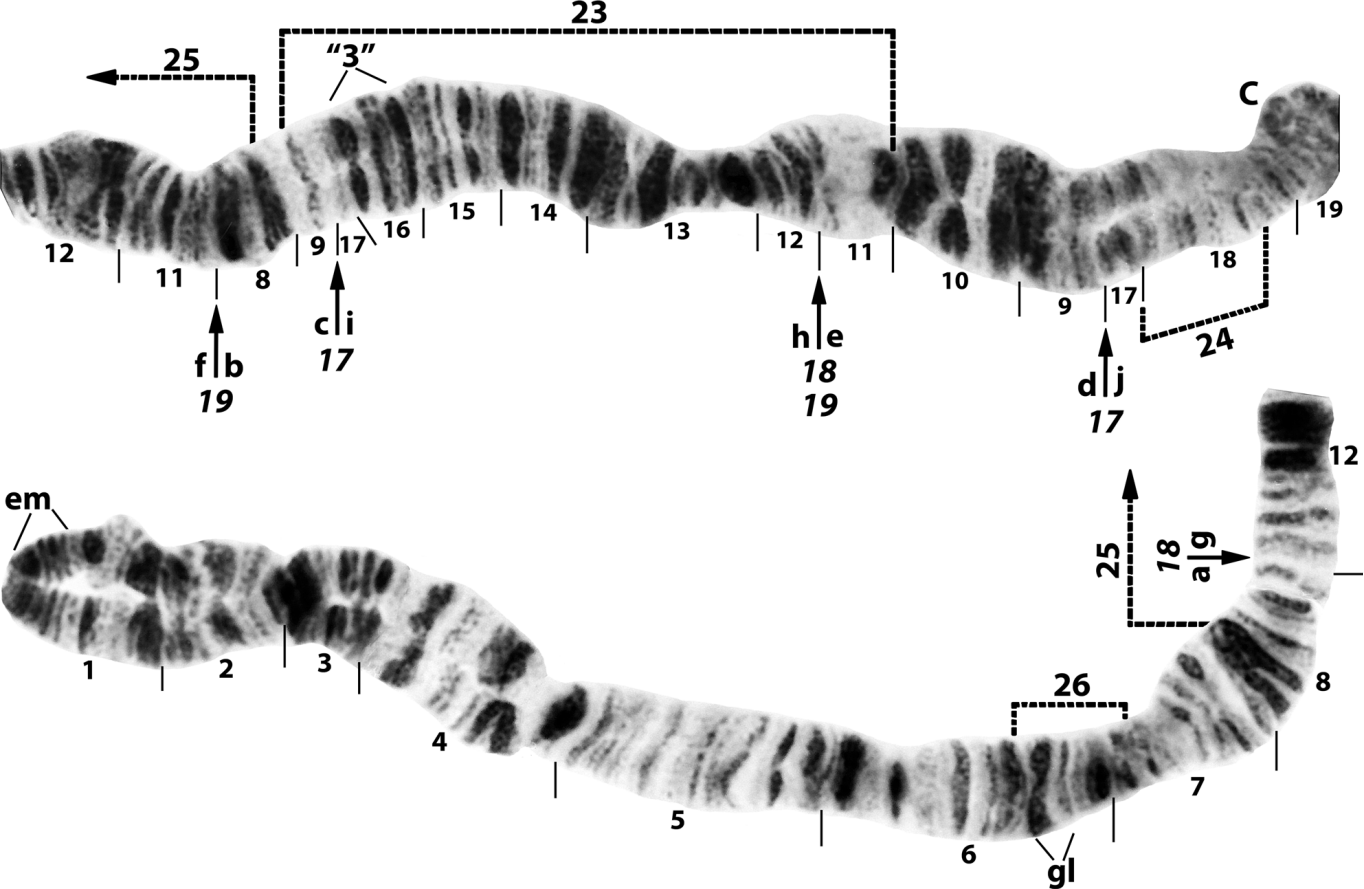
IS arm of *Simulium nigricoxum* (female larva) from Canada, Thelon River, showing the *IS-17*,*18*,*19* sequence. Breakpoints of fixed inversions are indicated by arrows. Limits of Y-linked IS-23, IS-24, IS-25, and IS-26 are indicated by dotted brackets. The standard sequence for the subgenus *Simulium* can be obtained from the *IS-17*,*18*,*19* sequence by alphabetically ordering fragments indicated by small letters ‘a’ through ‘j’. *IS-18* and *IS-19* share one coincident breakpoint. C = centromere, em = end marker, gl = glazed, “3” = 3 marker.

**Fig 2:**
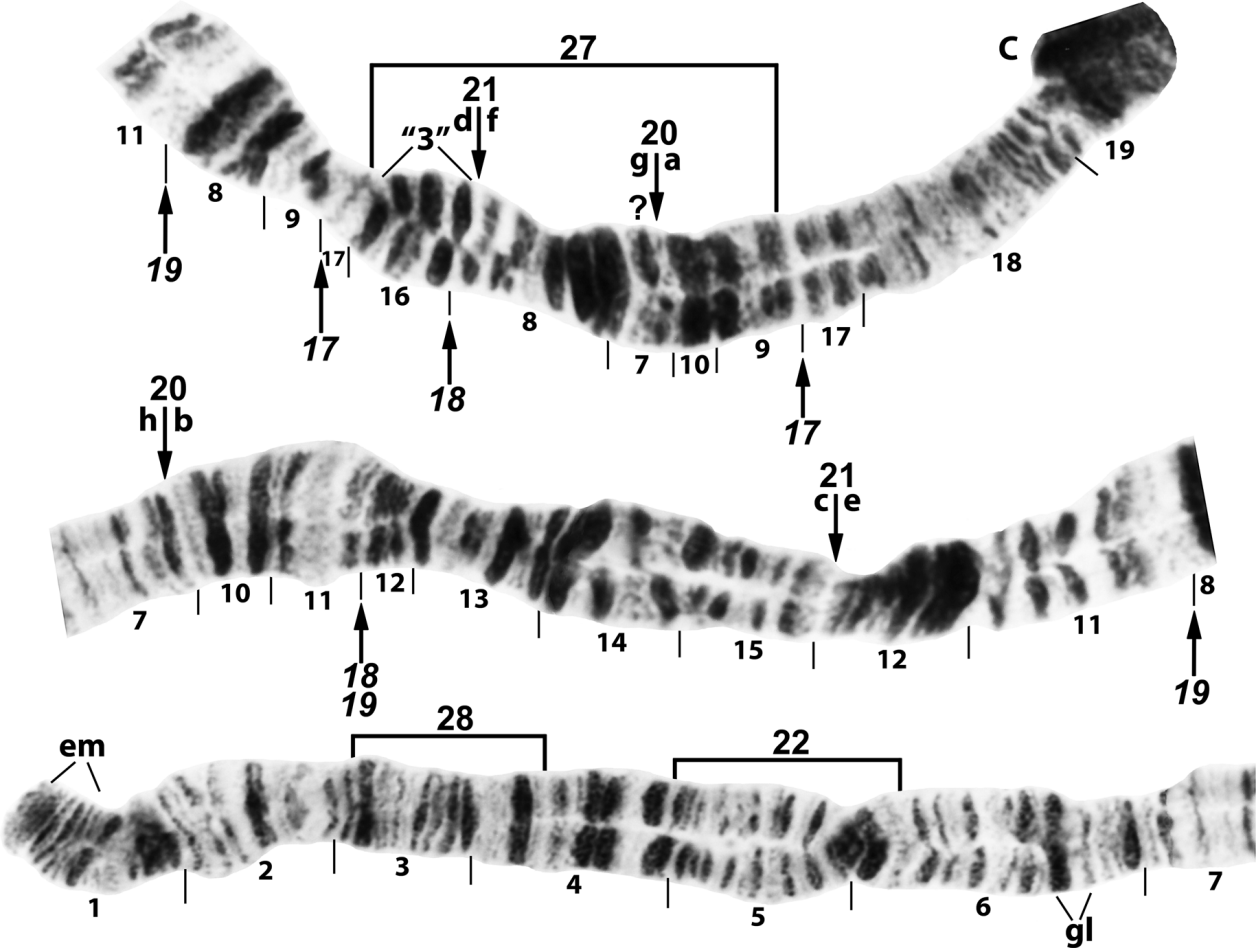
IS arm of *Simulium cholodkovskii* (female larva), showing the *IS-17*,*18*,*19*,20,21 sequence. Fixed inversions *IS-17*,*18*,*19* are indicated with arrows below the chromosome, and predominant polymorphic inversions IS-20 and IS-21 with arrows above the chromosome; the basic *IS-17*,*18*,*19* sequence can be obtained from the *IS-17*,*18*,*19*,20,21 sequence by alphabetically ordering the fragments indicated by small letters ‘a’ through ‘h’. Limits of polymorphic inversions IS-22, IS-27, and IS-28 are indicated by brackets. C = centromere, em = end marker, gl = glazed, “3” = 3 marker;? = band unaccounted for but attributed to section 7.

**Fig 3:**
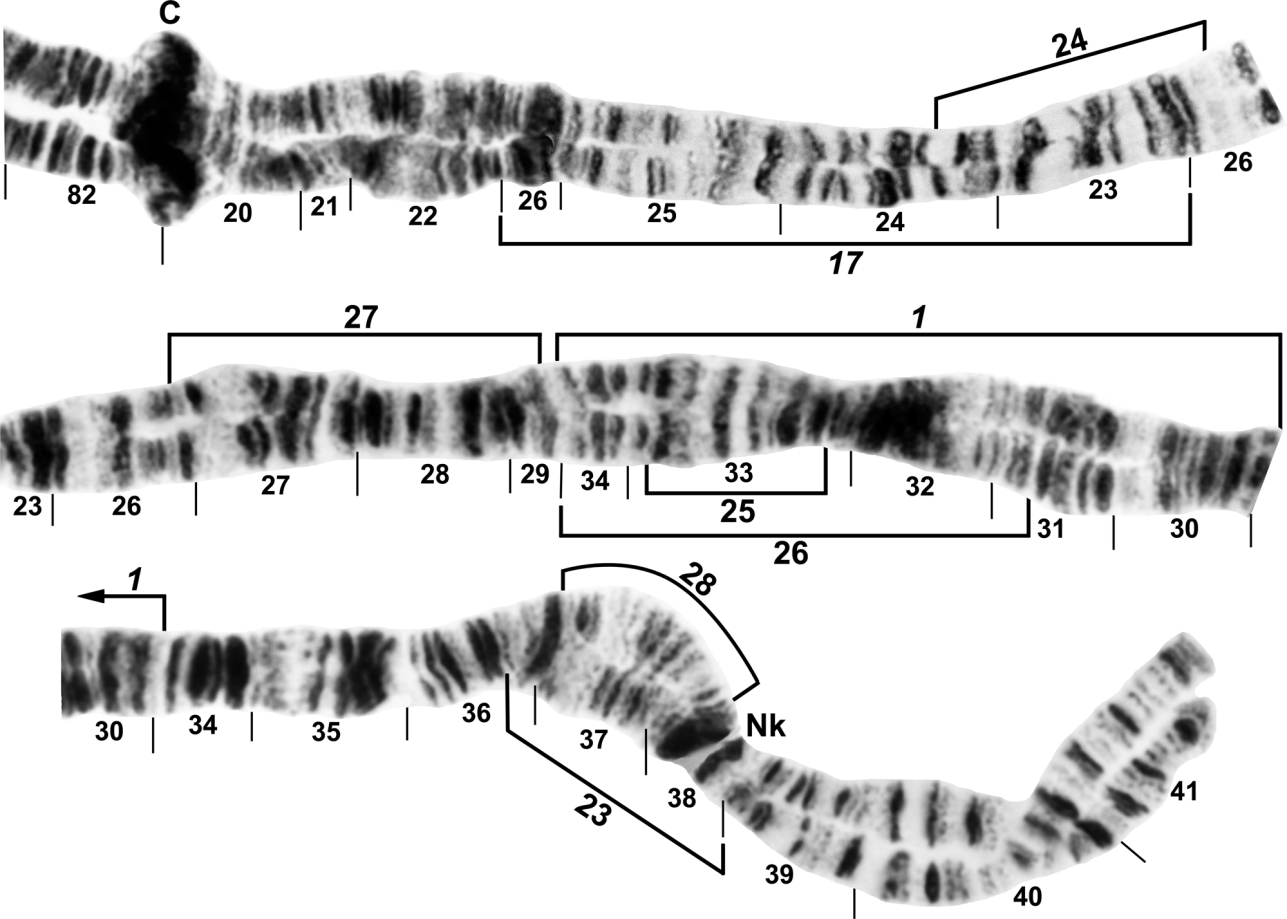
IL arm of *Simulium cholodkovskii*, showing continuity of transposed arms IIIS and IL and the *IL-1*,*17* sequence. The map represents a composite male (sections 23–25) and female larva. Limits of polymorphic inversions IL-23–IL-28 are indicated by brackets. C = centromere, Nk = neck.

**Fig 4:**
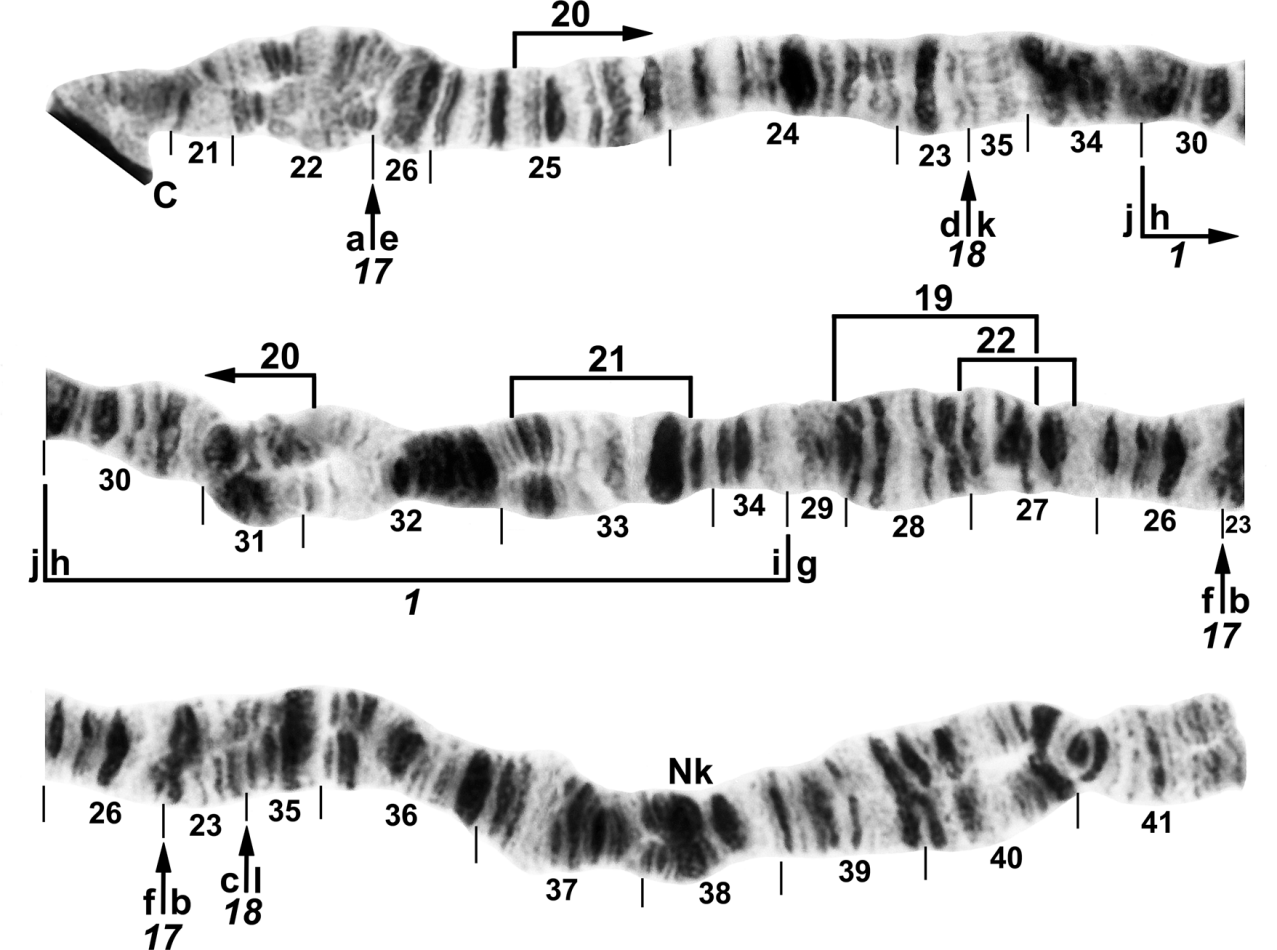
IL arm of *Simulium nigricoxum* (female larva) from Canada, Thelon River, showing the *IL-1*,*17*,*18* sequence. The 3 fixed inversions are shown by a bracket (*IL-1*) and arrows (*IL-17*,*18*). The standard sequence for the subgenus *Simulium* can be obtained from the *IL-1*,*17*,*18* sequence by alphabetically ordering the fragments indicated by small letters ‘a’ through ‘l’. Limits of polymorphic inversions IL-19–IL-22 are indicated by brackets. C = centromere, Nk = neck.

**Fig 5:**
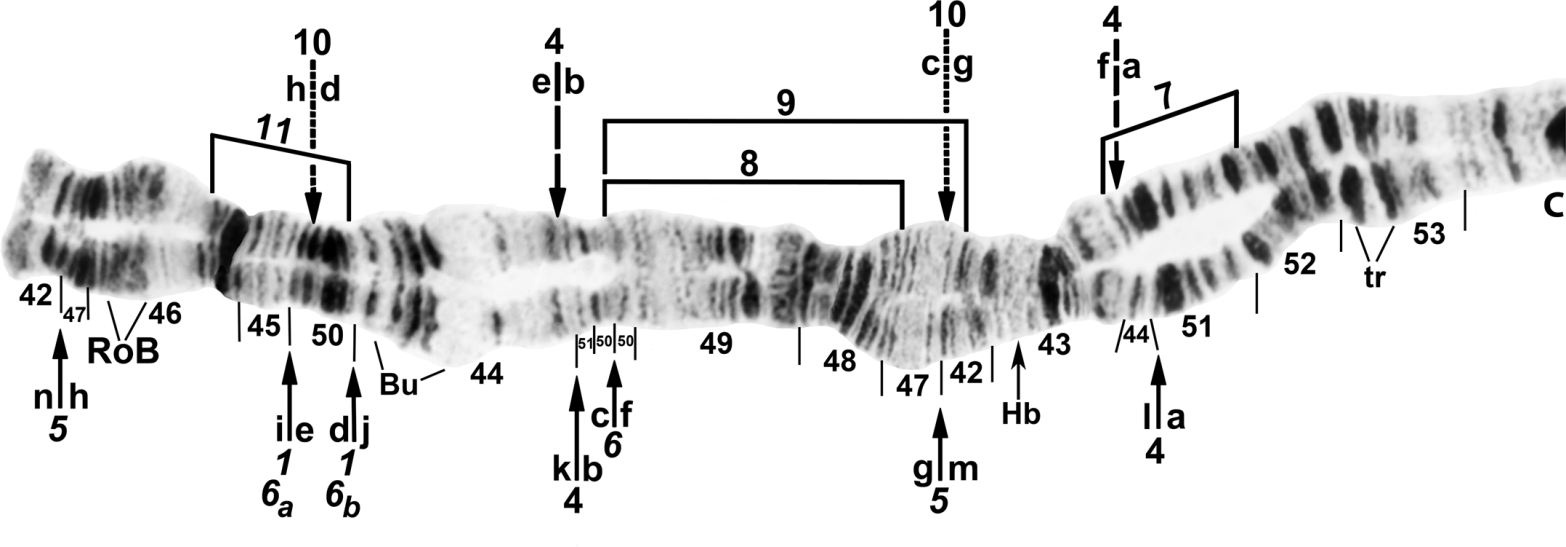
IIS arm of *Simulium cholodkovskii* (female larva), showing the typical sequence (*IIS-1*,4,*5*,*6*). Limits of polymorphic inversions IIS-7–IIS-9 and IIS-11 are indicated by brackets; *6*_*a*_ and *6*_*b*_ denote alternative breakpoints for *IIS-6*. The standard sequence for the subgenus *Simulium* can be obtained from the *IIS-1*,4,*5*,*6* sequence by alphabetically ordering the fragments indicated by small letters ‘a’ through ‘n’ that appear below the chromosome. Ordering the fragments above the chromosome from ‘a’ to ‘h’ produces the Y-chromosome sequence (*IIS-1*,*5*,*6*,10) of *S*. *nigricoxum*, i.e. IIS-10 is Y linked (dotted lines), whereas IIS-4 is X-linked (dashed lines) and, therefore, absent on the Y. Bu = bulge, C = centromere, Hb = location of heteroband 43Hb, RoB = ring of Balbiani, tr = trapezoidal.

**Fig 6:**
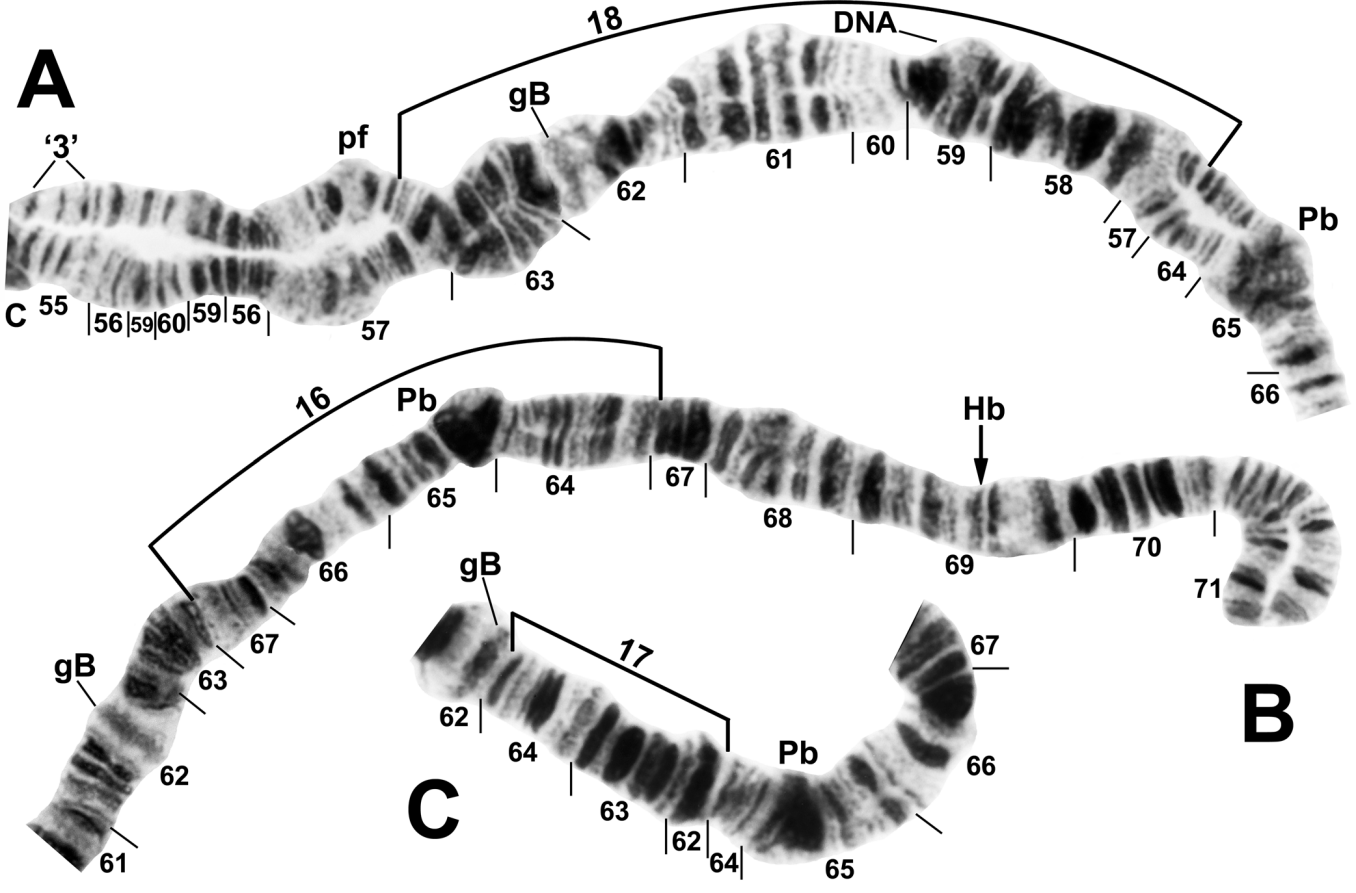
IIL arm of the *Simulium cholodkovskii* lineage. A. *Simulium decimatum* (female larva), showing the *IIL-1*,*2*,18 sequence. *IIL-1*,*2* are not bracketed but the renumbered sections (56–60) in the base of the arm indicate their presence. B. *Simulium cholodkovskii* (composite female + male larva [proximal 5 sections]), showing the IIL-16 sequence. C. *Simulium cholodkovskii* (female larva), showing the IIL-17 sequence. C = centromere, DNA = DNA puff, gB = gray band, Hb = location of heteroband 69Hb, Pb = parabalbiani, pf = puffing band, ‘3’ = 3 sharp.

**Fig 7:**
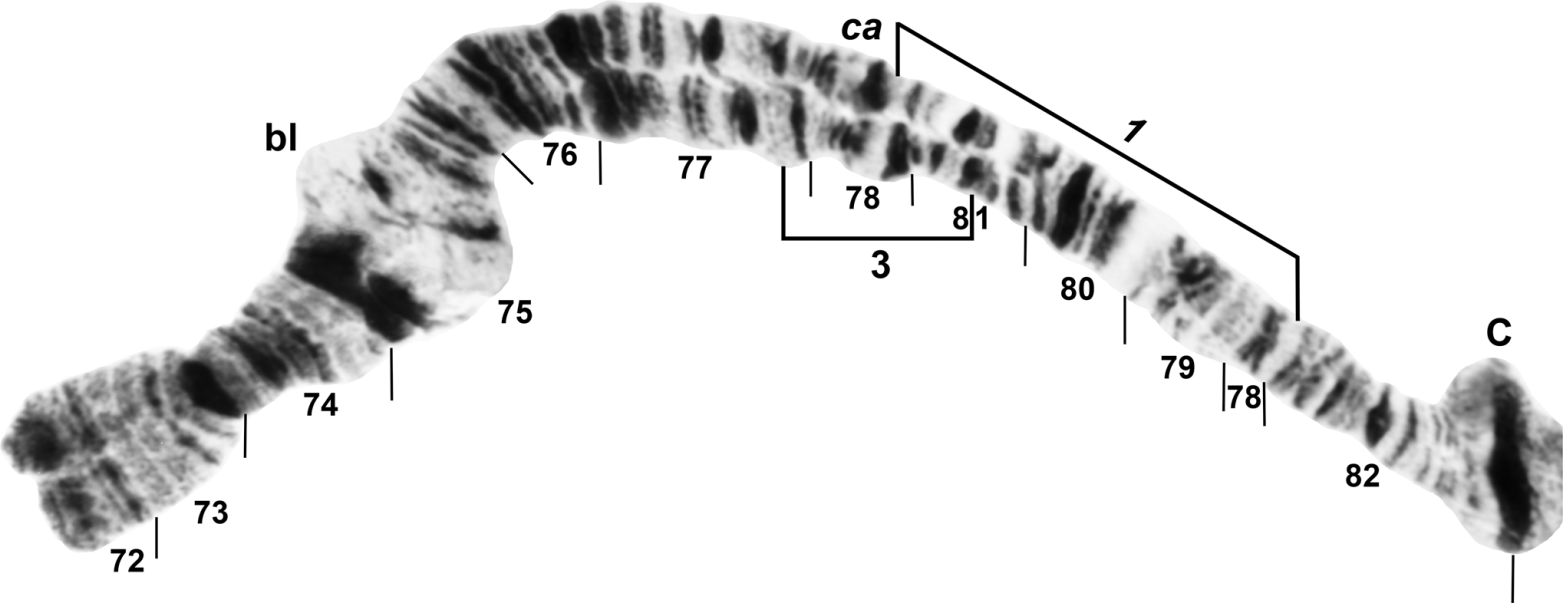
IIIS arm of *Simulium cholodkovskii* (female larva), showing the *IIIS-1* sequence. Limits of polymorphic inversion IIIS-3 are shown by a bracket; bl = blister, C = centromere, ca = capsule.

**Fig 8:**
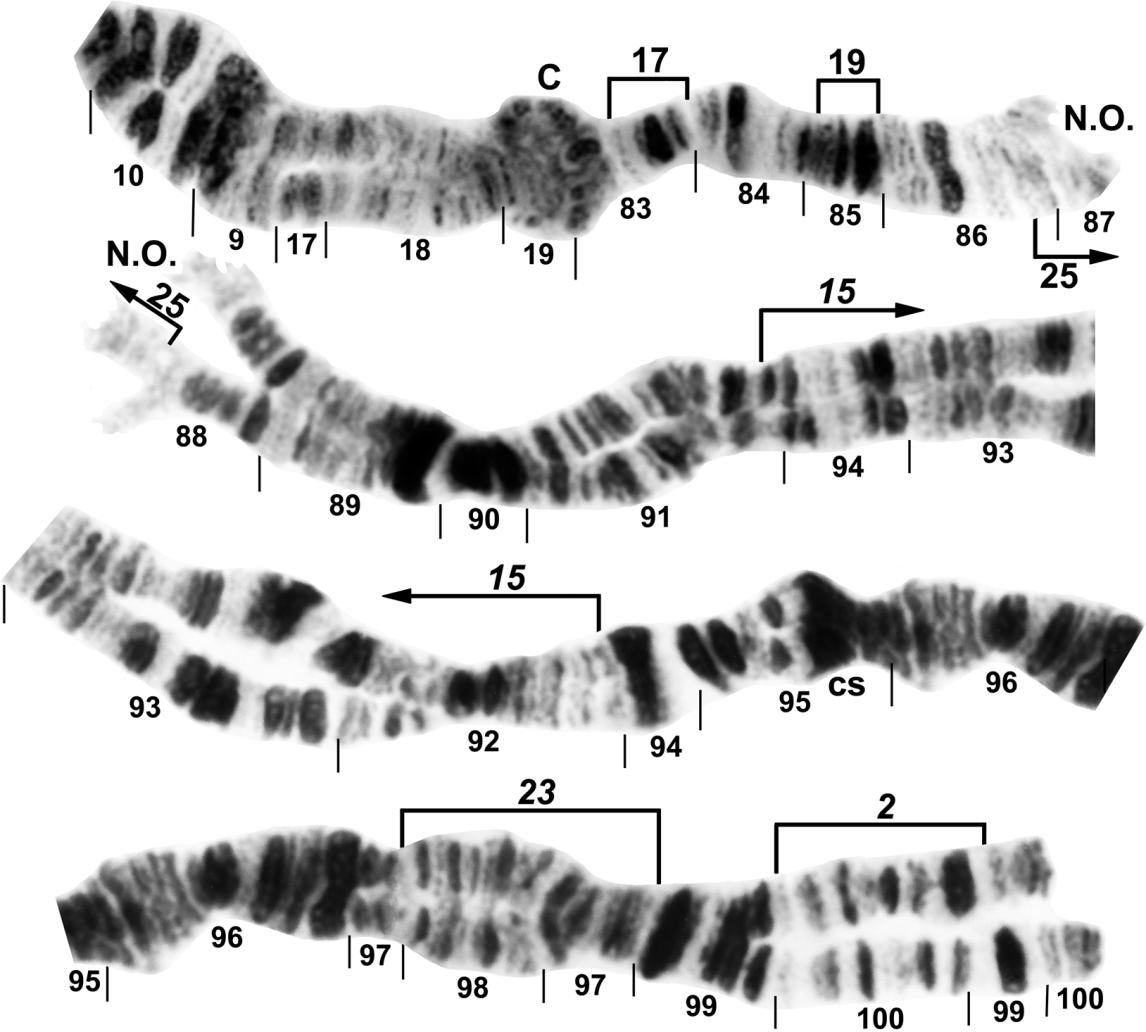
IIIL arm of *Simulium decimatum*, showing continuity of transposed arms IS and IIIL and the *IIIL-2*,*15*,*23* sequence. The map represents a composite male (sections 98–100) and female larva, with sections 10–87 from the Thelon River and the remainder from the Tuul River. Polymorphic inversion IIIL-17, IIIL-19, and IIIL-25 (shared with *S*. *cholodkovskii*) are indicated by brackets. C = centromere, cs = cup and saucer marker, N.O. = nucleolar organizer.

**Fig 9:**
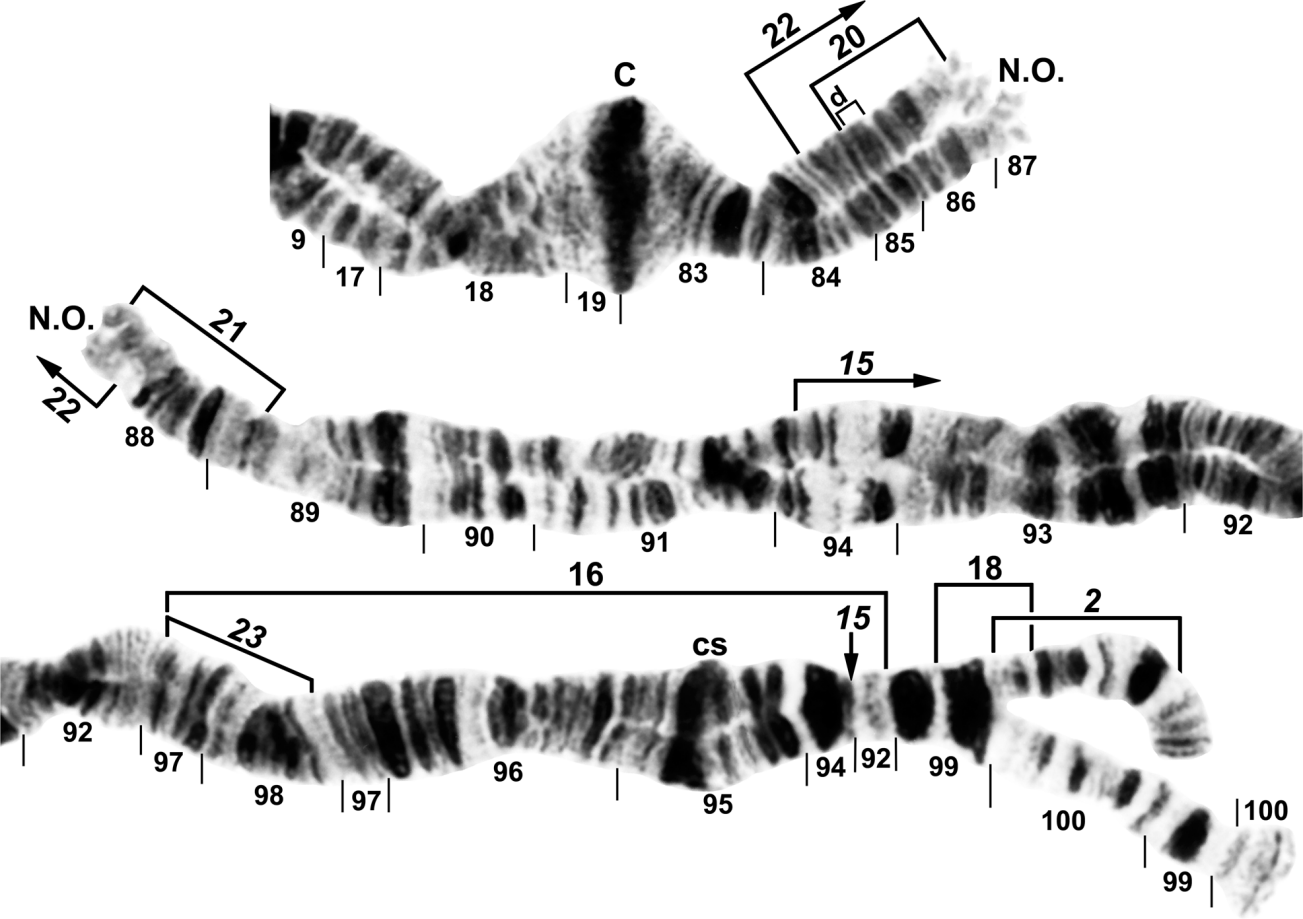
IIIL arm of *Simulium cholodkovskii*, showing continuity of transposed arms IS and IIIL and the *IIIL-2*,*15*,16,*23* sequence. The map represents a composite male (sections 95 middle–100) and female larva. Polymorphic inversions IIIL-18, IIIL-20, IIIL-21, and IIIL-22 are indicated by brackets. C = centromere, cs = cup and saucer marker, d = location of deletion of 2 bands in section 85, N.O. = nucleolar organizer.

All larvae had the typical *n* = 3 haploid complement. Homologues were tightly paired. Centromere bands were each in the middle of a variously expanded centric region (Figs 1–9). Supernumerary chromosomes were absent. Each chromosome arm was recognized by one or more landmarks. The short arm of chromosome one (IS) had the diagnostic ‘end marker’, a terminal series of evenly spaced, fine bands (Figs 1 and 2). IL had the distal ‘neck’, only rarely redistributed by an inversion (Figs 3 and 4). IIS was characterized by the ‘bulges’ and the subterminal ‘ring of Balbiani’, a conspicuous marker present at the family level (Fig 5). IIL had a number of markers, particularly the ‘parabalbiani’, which was mobile within the arm (Fig 6). IIIS, the shortest arm, was characterized by the distal ‘blister’ marker (Fig 7), and IIIL by its basal nucleolar organizer at the junction of sections 87 and 88 (Figs 8 and 9).

All larvae were fixed for a whole-arm interchange between chromosomes I and III, producing the combination IS + IIIL, IL + IIIS. The longest of the 3 submetacentric chromosomes was IS + IIIL. No microscopic banding differences in the centromeric regions could be resolved between members of the *S*. *cholodkovskii* lineage and the *Simulium* subgeneric standard or an extant, non-translocation relative (*S*. *malyschevi*). Hence, determination of breakpoints was equivocal with respect to whether they were proximal or distal between chromosomes I and III in the derivation of the translocation homozygote (Figs 3 and 9).

In addition to the translocation, the basic sequence common to all larvae differed from the subgeneric standard sequence by 14 fixed inversions: *IS-17*,*18*,*19*; *IL-1*,*17*; *IIS-1*,*5*,6*; IIL-1*,*2*; *IIIS-1*; and *IIIL-2*,*15*,*23*. *IS-18* and *IS-19* shared one coincident breakpoint and *IS-18* and the autosomal polymorphism IS-21 shared another. Our interpretation of the IIS sequence (Fig 5) represents the most parsimonious hypothesis of inversion breakpoints, and was based on the constraint that the *IIS-1* sequence—shared by all analyzed members of the *S*. *malyschevi* and *S*. *reptans* groups [28]—was represented as the first inversion in the derivation from the subgeneric standard. We interpreted one of the breakpoints of *IIS-6* as coincident with one or the other of the *IIS-1* breakpoints, although which of the two breakpoints could not be determined; therefore, both possibilities for the coincident break (*IIS-6*_*a*_ and *IIS-6*_*b*_) are depicted on our map (Fig 5).

Additional rearrangements permitted subdivision of our material into three chromosomally defined taxa that we designated *S*. *cholodkovskii*, *S*. *decimatum*, and *S*. *nigricoxum*.

### Simulium cholodkovskii

Larvae (*n* = 90) of *S*. *cholodkovskii* from Mongolia’s Tuul River (Sites 1 and 2) exhibited ectopic pairing of heavily stained centromere bands and were fixed for *IIS-4* (Fig 5) and nearly fixed (frequency > 0.96) for IS-20,21 (Fig 2) and IIIL-16 (Fig 9). Our interpretation of the predominant IS sequence (*IS-17*,*18*,*19*,20,21) left one band at the junction of sections 7 and 10 unattributed to its original section (query mark in Fig 2); we tentatively ascribed it to section 7. IS-21 was included entirely within the much larger IS-20; the two inversions were absolutely linked, never occurring independently. Inversions IIL-16 and IIL-17 together accounted for 97% of the IIL sequences and IIL-18 the remainder (Fig 6), except in one male that had a IIL-17 homologue paired with a standard homologue. IS-22, a common autosomal polymorphism unique to *S*. *cholodkovskii*, was in Hardy-Weinberg equilibrium (Table 2). Twenty-four additional autosomal inversions were in low (< 0.06) overall frequency (Table 2). The sex chromosomes were microscopically undifferentiated (X_0_Y_0_).

**Table 2:** Frequency of rearranged constituents for all chromosomal rearrangements in the *Simulium cholodkovskii* lineage, relative to the *Simulium* subgeneric standard.

	*S*. *cholodkovskii*		*S*. *decimatum*		*S*. *nigricoxum*
	Site 1	Site 2	Site 1	Site 2	Site 3
Females:Males	38:32	12:8	7:13	3:3	12:10
*IS + IIIL*, *IL + IIIS*	1.00	1.00	1.00	1.00	1.00
*IS-17*	1.00	1.00	1.00	1.00	1.00
*IS-18*	1.00	1.00	1.00	1.00	1.00
*IS-19*	1.00	1.00	1.00	1.00	1.00
IS-20	0.97	0.95	0.02		
IS-21	0.97	0.95	0.02		
IS-22	0.34^1^	0.22			
IS-23			*^2^	**^3^	
IS-24			*^2^	**^3^	
IS-25			*^2^		
IS-26				**^3^	
IS-27		0.02			
IS-28	0.01				
*IL-1*	1.00	1.00	1.00	1.00	1.00
*IL-17*	1.00	1.00	1.00	1.00	1.00
*IL-18*					1.00
*IL-19*				0.08	
IL-20	<0.01		0.02		
IL-21			0.02		
IL-22	<0.01				
IL-23	0.01				
IL-24	<0.01				
IL-25	<0.01				
IL-26	<0.01				
IL-27	0.03				
IL-28	<0.01				
*IIS-1*	1.00	1.00	1.00	1.00	1.00
IIS-4	1.00	1.00	1.00	1.00	***^4^
*IIS-5*	1.00	1.00	1.00	1.00	1.00
*IIS-6*	1.00	1.00	1.00	1.00	1.00
IIS-7			0.88	0.83	
IIS-8	0.02				
IIS-9	<0.01				
IIS-10					***^5^
IIS-11	0.01	0.02			
IIS 43Hb	<0.01				
*IIL-1*	1.00	1.00	1.00	1.00	1.00
*IIL-2*	1.00	1.00	1.00	1.00	1.00
IIL-16	0.74	0.70	0.05	0.08	
IIL-17	0.23	0.25	0.02		
IIL-18	0.03	0.05	0.92	0.92	
IIL 69Hb			0.02		
*IIIS-1*	1.00	1.00	1.00	1.00	1.00
IIIS-3	<0.01				
*IIIL-2*	1.00	1.00	1.00	1.00	1.00
*IIIL-15*	1.00	1.00	1.00	1.00	1.00
IIIL-16	0.96	1.00			
IIIL-17			0.02		
IIIL-18	<0.01				
IIIL-19				0.17	
IIIL-20	0.01	0.02			
IIIL-21	<0.01	0.02			
IIIL-22	<0.01		0.02		
*IIIL-23*	1.00	1.00	1.00	1.00	1.00
IIIL-25	0.07		0.05		
IIIL-24	<0.01				
IIIL 85d^6^			0.02		
IIIL repattern^7^	0.01				

Sites: 1, 2 (Mongolia), 3 (Canada).

^1^ IS-22 was in Hardy-Weinberg equilibrium (ss = 29, si = 34, ii = 7, where s = standard, i = inverted; χ^2^ = 0.42, df = 1, P > 0.05).

^2^ * IS-23, IS-24, and IS-25 were linked to the Y chromosome: 4 males had undifferentiated sex chromosomes (X_0_Y_0_), 4 were heterozygous for IS-23 (X_0_Y_1_), 3 were heterozygous for IS-23,24 (X_0_Y_2_), and 1 was heterozygous for IS-23,24,25 (X_0_Y_3_). An additional male was heterozygous for IS-20,21, although whether these inversions were on the X or the Y homologue could not be determined; the other homologue had only the typical sequence for the lineage (i.e., *IS-17*,*18*,*19*). One female was heterozygous for IS-23 (X_0_ X_1_); the other 6 females had only the typical *IS-17*,*18*,*19* sequence (X_0_ X_0_).

^3^ ** IS-23, IS-24, and IS-26 were linked to the Y chromosome: 1 male was heterozygous for IS-23 (X_0_Y_1_), 1 was heterozygous for IS-23,24 (X_0_Y_2_), and 1 was heterozygous for IS-23,24,26 (X_0_Y_4_). All 3 females lacked IS-23, IS-24, and IS-26 (X_0_X_0_).

^4^ *** IIS-4 was linked completely to the X chromosome (X_1_).

^5^ *** IIS-10 was linked completely to the Y chromosome (Y_1_).

^6^ 85d = deletion of 2 bands in section 85 (Fig 9).

^7^ 1 female and 1 male had heterozygous repatterning of bands, including additional bands, in sections 85–87 that could not be accounted for by inversions.

### Simulium decimatum

Larvae (*n* = 26) of *S*. *decimatum* were found at the same sites (1 and 2) as *S*. *cholodkovskii* in Mongolia’s Tuul River. The two species had the same ectopic pairing of heavily stained centromere bands and fixation of *IIS-4*. No fixed inversions distinguished *S*. *decimatum* from *S*. *cholodkovskii*, although the preponderant sequences in each of the two species, including two nearly fixed-inversion differences, allowed absolute distinction. IIIL-16 was entirely absent from *S*. *decimatum*, and IS-20,21 (frequency = 0.02) nearly so. IIL-18 (Fig 6A) was the predominant IIL sequence (0.92), and IIL-16 and IIL-17 were in low frequency (0.06) in *S*. *decimatum*. IIS-7 (Fig 5) was frequent (0.87) and exclusive to *S*. *decimatum*. Nine additional autosomal inversions were in low (< 0.04) overall frequency (Table 2). Of 16 males at the two sites, either the sex chromosomes were undifferentiated (X_0_Y_0_, 25.0%) or the Y was indicated by linkage of IS-23 alone (X_0_Y_1_, 31.2%), in combination with IS-24 (25.0%, X_0_Y_2_), or once each with IS-25 (6.2%, X_0_Y_3_) and IS-26 (6.2%, X_0_Y_4_) (Table 2, Fig 1). No crossover types were seen. One additional male had IS-20,21 on one homologue and no heterozygous inversions on the opposite homologue; neither homologue could be associated with the X or the Y. Nine of the 10 females were X_0_X_0_, and one female (X_0_X_1_) carried IS-23 heterozygously. Consequently, IS-23 demarked both a genetic X and Y.

### Simulium nigricoxum

Larvae (*n* = 22) from Canada’s Thelon River (Site 3) had the same basic sequence as *S*. *decimatum* but were uniquely fixed for *IL-18* (Fig 4). Unlike *S*. *decimatum*, *S*. *nigricoxum* lacked IIS-7 and IIL-18 and was autosomally monomorphic. Centromere bands were less heavily stained than in the other two taxa and ectopic pairing was absent. Sex determination was based on the IIS arm. X_1_ was characterized by IIS-4 and the standard sequence for IIS-10, whereas Y_1_ was characterized by IIS-10 and the standard sequence for IIS-4. All females were X_1_X_1_ and all males X_1_Y_1_. The X sequence, therefore, was *IIS-1*,4,*5*,*6*. We interpreted the Y chromosome as two inversions removed from the X sequence. That is, IIS-10 was Y-linked and IIS-4 was absent, yielding the *IIS-1*,*5*,*6*,10 sequence (Fig 5), relative to the subgeneric standard. Thus, males were double heterozygotes. Several breakpoints of the X and Y sequences, relative to the subgeneric standard, differed by one or two fine bands, complicating interpretations; our hypothesis for these breakpoints is shown in Fig 5.

### Origin and Fixation of the Interchange, with Subsequent Speciation

**Fig 10:**
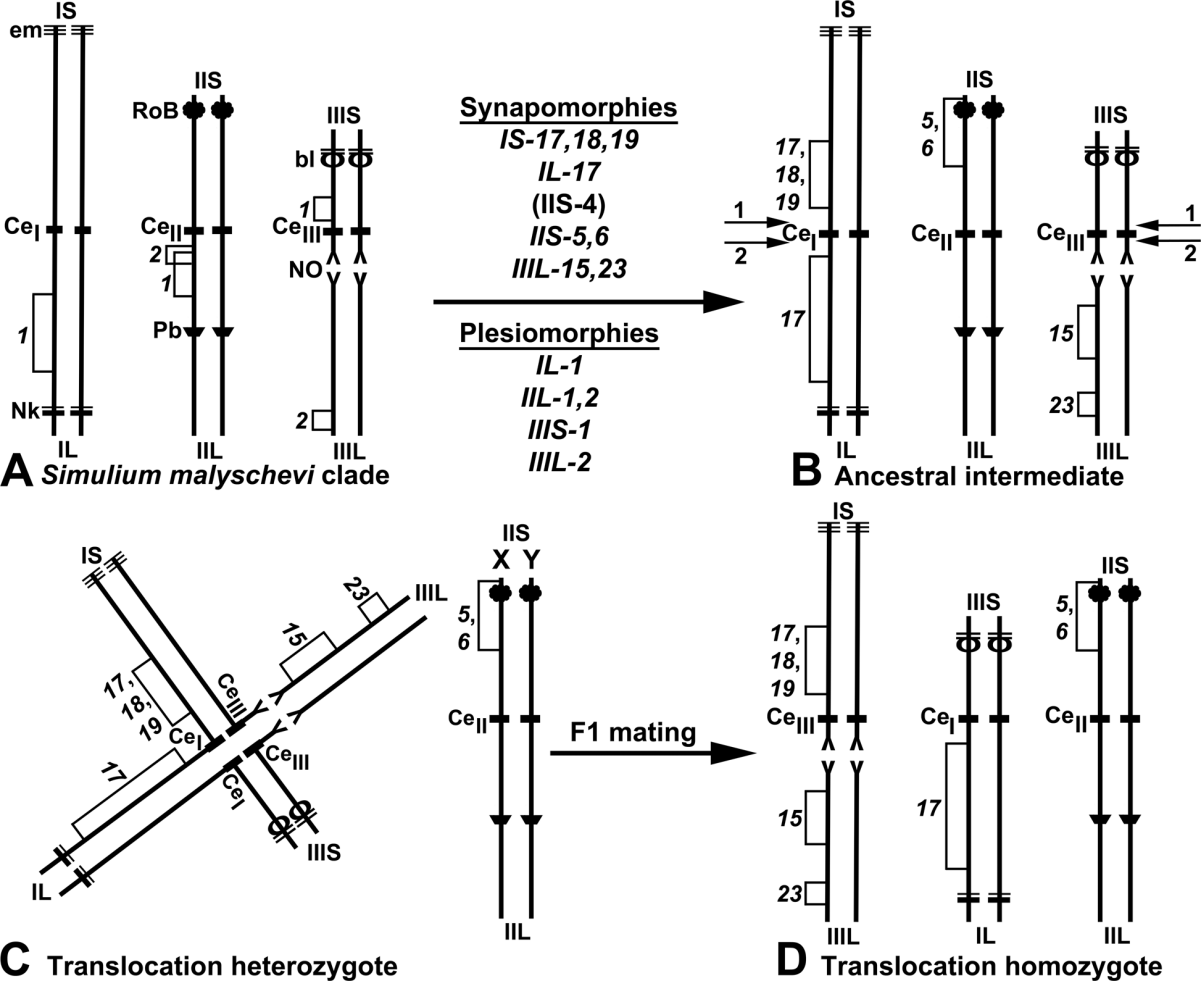
Schematic derivation of the monocentric translocation homozygotes that define the *Simulium cholodkovskii* lineage. Diagnostic landmarks are given on idiograms for short (S) and long (L) arms of the three chromosomes (I, II, III). Both homologues of each chromosome are shown; bl = blister with 2 heavy bands, Ce = centric region containing putative centromere, em = end marker, Nk = neck, NO = nuclear organizer, Pb = parabalbiani, RoB = ring of Balbiani. Fixed inversions are italicized and bracketed on the left side of each chromosome; polymorphic inversions are in standard type and bracketed on the right side. A. Standard chromosomal complement, showing characteristic inversions of the *Simulium malyschevi* clade (*IL-1*, *IIL-1*, *IIL-2*, *IIIS-1*, and *IIIL-2*) from which the sequences of the *S*. *cholodkovskii* lineage are derived. From the ancestral intermediate through present-day members of the lineage (B–D), these inversions carry through as plesiomorphies, but are not shown in subsequent idiograms. B. Hypothetical intermediate of the *Simulium cholodkovskii* lineage, with characteristic fixed inversions established before the translocation event. Arrows 1 and 2 represent proximal and distal breaks, respectively, in the centric regions of chromosomes I and III. C. Derivation of a monocentric translocation heterozygote expressed as one of two possible scenarios: 1) As shown, chromosomal breaks occur in the proximal centric regions of chromosomes I and III (arrows 1 in Fig 10B) such that IS joins with CeIII plus IIIL and IL plus CeI joins with IIIS, giving rise to translocation heterozygote progeny (first generation). 2) (Not shown) chromosomal breaks occur in the distal centric regions of chromosomes I and III (arrows 2 in Fig 10B) such that IS plus CeI joins with IIIL and IL joins with CeIII plus IIIS. IIS is shown as the putative sex arm (X, Y). D. Monocentric translocation homozygote formed from an F1 mating. In our model, the first appearance of translocation homozygotes occurs in the F2 as a result of matings between F1 translocation males and females.

Evolution of the *S*. *cholodkovskii* lineage and derivation of the monocentric translocation homozygotes that define the lineage proceeded from the *S*. *malyschevi* group, which is characterized by inversions *IL-1*, *IIL-1*, *IIL-2*, *IIIS-1*, and *IIIL-2* (Fig 10A), through the hypothetical ancestral intermediate of the *S*. *cholodkovskii* lineage, which we depict as carrying synapomorphic fixed inversions *IS-17*,*18*,*19*; *IL-17*; and *IIIL-15*,*23* (Fig 10B). Although we show these inversions in the ancestral intermediate, rather than subsequent to the translocation event, evidence favoring one occurrence before the other is lacking.

A major step in the evolution of the *S*. *cholodkovskii* lineage was the derivation of a monocentric heterozygote via one of two possible scenarios. One scenario might have involved double-strand chromosomal breaks in the proximal centric regions of chromosomes I and III, such that IS joined with CeIII plus IIIL, and IL plus CeI joined with IIIS, giving rise to translocation heterozygote progeny (Fig 10C). A second possibility would have involved chromosomal breaks in the distal centric regions of chromosomes I and III, such that IS plus CeI joined with IIIL, and IL joined with CeIII plus IIIS. II is regarded as the putative sex chromosome in the ancestral intermediate and, thus, in the translocation heterozygote (Fig 10C). The final, major step involved formation of translocation homozygotes, assuming alternate disjunction of translocation heterozygotes, assortative mating, and a selective advantage of the translocation homozygotes in the initial population (F2 second generation) (Fig 10D).

These events would proceed via the following scenario. Fidelity of alternate reductional segregation of the translocated chromosomes at anaphase one (AI) to the same pole, and the typical (non-translocated) chromosomes to the other pole, would result in two of the four anaphase cells (AII) having the translocated karyotype through equational division and the other two cells having the non-translocated complement. Assuming random segregation of chromosome II, the putative sex chromosome, 50% of the cells (i.e., spermatozoa) would have a genetic X, and 50% would have a genetic Y. Heterozygous males would produce sperm with four different genotypes: translocation complement plus Y, typical complement plus Y, translocation complement plus X, and typical complement plus X. If these males mated with typical females, they would produce F1 heterozygous males and females. Adult F1 females would produce eggs with two different genotypes: translocation complement plus X, and typical complement plus X, with the caveat that only one of the four meiotic products would become the functional egg during oogenesis. Heterozygous translocation adults from this F1 population (via sibling matings) would produce F2 offspring in the following ratio: 1 typical complement: 2 translocation heterozygotes: 1 translocation homozygote. Once the translocation was established, subsequent speciation would involve further structural differentiation of the complement, primarily involving inversions, resulting in chromosomally distinct species.

### Phylogenetic Relationships

**Fig 11:**
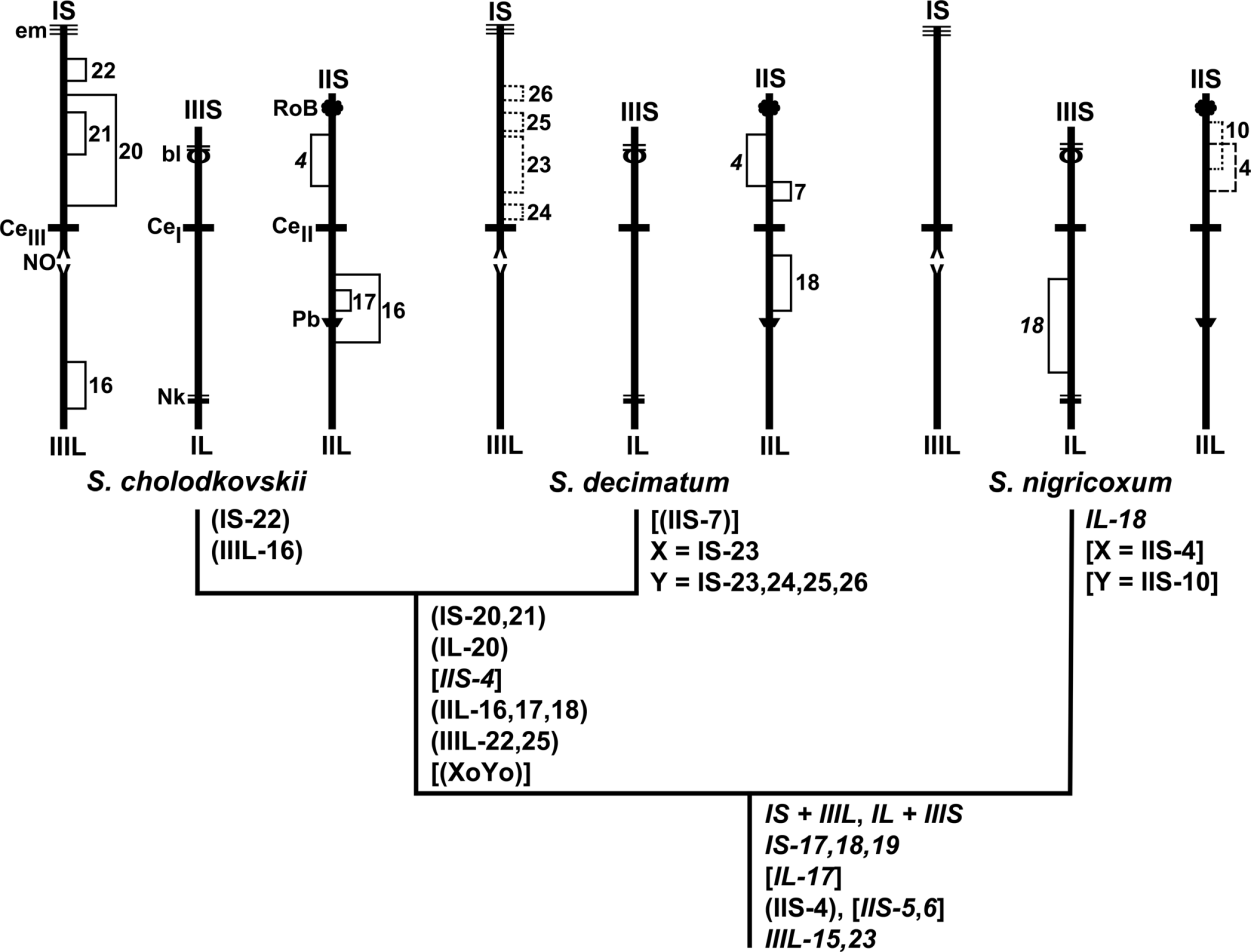
Cytophylogeny of the *Simulium cholodkovskii* lineage, with terminals depicted by idiograms of each species. The outgroups (*S*. *erythrocephalum* and *S*. *vittatum*) are not shown on the cladogram. Rearrangements are shown in italics if fixed synapomorphies, in parentheses if polymorphic synapomorphies, and in square brackets if shared characters that could not be determined as plesiomorphic or synapomorphic. The homologues of each chromosome (I, II, III) are shown as tightly synapsed, and the arms are indicated as long (L) or short (S). Only diagnostic inversions (frequency > 0.10) for each species are shown on the idiograms. Fixed inversions are bracketed on the left. Polymorphic inversions are bracketed on the right as solid lines if autosomal, dashed if X-linked, and dotted if Y-linked. Landmarks are labeled on the idiogram for *S*. *cholodkovskii*: bl = blister with 2 heavy bands, Ce = centric region (with subscript indicating chromosome I, II, or III), em = end marker, Nk = neck, NO = nucleolar organizer, Pb = parabalbiani, RoB = ring of Balbiani.

Outgroup comparisons allowed us to determine that all IS, IIL, IIIS, and IIIL rearrangements were synapomorphic. That is, in addition to the interchange, *IS-17*,*18*,*19* and *IIIL-15*,*23* were uniquely shared characters (inversions) derived from a common ancestor of the *S*. *cholodkovskii* lineage (Fig 11). IIS-4 is shown as polymorphic in the ancestor to account for its different fates—fixed in *S*. *cholodkovskii* and *S*. *decimatum* and X-linked in *S*. *nigricoxum*. *IL-1*; *IIS-1*; *IIL-1*,*2; IIIS-1*; and *IIIL-2* are common to a larger clade that includes the *S*. *malyschevi* group, of which the *S*. *cholodkovskii* lineage is a part [28]. IIIL-16 was unique to *S*. *cholodkovskii*. The Y-linked inversions in IS uniquely defined *S*. *decimatum*. Other inversions (e.g., *IL-18*, IIS-7, IIS-10) that were found in only one of our three taxa also might be uniquely derived, but we were not able to reconcile the polarity of these sequences with that in both outgroups; however, we determined that *IL-18* was uniquely derived, relative to the sequence in one of the two outgroups (*S*. *erythrocephalum*). By virtue of eight shared polymorphisms (Table 2, Fig 11), *S*. *cholodkovskii* and *S*. *decimatum* are sister species; the caveat, however, is that one or the other of the two taxa had each polymorphism in low frequency (< 0.09), suggesting that introgression, rather than common ancestry, is an equally plausible explanation for the uniquely shared polymorphisms.

## Discussion

### Taxonomic and Phylogenetic Inferences

All larvae can be assigned unequivocally to one of three chromosomal entities, based on a set of band sequences. Reproductive isolation can be shown in sympatry (Tuul River) for two of the chromosomal taxa—*S*. *cholodkovskii* and *S*. *decimatum*. If hybrids were present, inversions of high frequency (> 0.95) in *S*. *cholodkovskii* (i.e., IS-20,21 and IIIL-16) would be expected in some combination with inversions of high frequency (> 0.82) in *S*. *decimatum* (i.e., IIS-7 and IIL-18). Only a single partial example, a male heterozygous for IS-20,21; IIL-18; and IIS-7 but lacking IIIL-16, was found in one of 116 larvae. Of 56 fixed and polymorphic rearrangements in *S*. *cholodkovskii* and *S*. *decimatum*, relative to the subgeneric standard, 24 are common to both species, reflecting shared ancestry and perhaps some degree of introgression.

Given reproductive isolation of these two chromosomally distinct taxa, and the allopatric nature of the third chromosomal entity, what taxonomic conclusions can be drawn? Here, we are concerned with providing nomenclatural stability, recognizing that our applications of existing formal names to chromosomal segregates are hypotheses subject to further testing.

The type locality of *S*. *cholodkovskii* is the Uda River in Zabaikal Territory [29] about 420 km from our Site 1; along with Site 2, all three are in the Lake Baikal drainage. No geographic barriers to gene exchange separate the type locality from either Site 1 or Site 2. *Simulium cholodkovskii* has been recorded previously from the Tuul River by the original author (Rubtsov) of the species name [30]. We, therefore, consider our chromosomal characterization of *S*. *cholodkovskii* representative of the type specimen.

Our second chromosomal segregate is more problematic to associate with a formal name, and might represent any or several of the following six isomorphic possibilities: (1) *S*. *decimatum sensu stricto*, with the Ushakovka River (= Ida River) in Irkutskaya as its type locality [29] about 420 km from our Site 1; (2) *S*. *xerophilum* (Rubtsov), originally described as a subspecies of *S*. *decimatum* and now held in synonymy with *S*. *decimatum* but possibly a valid species with a type locality less than 100 km southeast of Sites 1 and 2; (3) *S*. *mongolicum* (Rubtsov), currently considered a distinct species, with its type locality within 300 km west of Sites 1 and 2; (4) *S*. *wagneri* Rubtsov, now in synonymy with *S*. *decimatum s*. *s*. and with its type locality an unspecified site in the Siberian Altai at least 1200 km to the west of our two Mongolian sites; (5) *S*. *yui* An & Yan, a synonym of *S*. *decimatum s*. *s*., described from Heilongjiang Province, China, more than 1200 km to the east; or (6) a previously unrecognized cryptic species. Given our inability to distinguish among these possibilities, the decision to apply the most familiar name, *S*. *decimatum*, to our chromosomal entity is a practical one, designed to provide the greatest nomenclatural stability and avoid, for example, recalling a dubiously applicable, obscure name from synonymy. Additional sampling throughout the area represented by the various type localities is needed for definitive resolution.

The species status of the third chromosomal segregate, *S*. *nigricoxum*, is not directly testable within the framework of the biological species concept. *Simulium nigricoxum* lies at the extreme eastern fringe of the range of the *S*. *cholodkovskii* lineage in the Western Hemisphere [20]. Although available evidence does not allow us to determine the geographic origin of the lineage or to date it, the connection of the Nearctic and Palearctic members of the lineage, as for many Holarctic simuliids, is through Beringia [20,27]. The tremendous geographic distance (> 7000 km) that separates *S*. *decimatum* and *S*. *nigricoxum* in Mongolia and Canada, respectively, reduces the question of species status to a pragmatic matter. Relative to more central populations of the *S*. *cholodkovskii* lineage, *S*. *nigricoxum* exhibits reduced chromosomal polymorphism characteristic of peripheral populations [31]. The existence of a unique fixed inversion (*IL-18*); absence of IIS-7, IIL-18, and ectopic pairing; and less heterochromatinization of centromere bands in *S*. *nigricoxum* provide some, albeit limited, rationale for recognizing a distinct species. Coupled with sex determination based on nonhomologous chromosomes (I in *S*. *decimatum*, II in *S*. *nigricoxum*), however, the argument for separate species acquires additional weight. Nonhomologous sex chromosomes typically are associated with different species [22,32].

A previously analyzed population of *S*. *nigricoxum* from the Canadian Yukon [23], about 1700 km directly west of our Canadian collection (Site 3), slightly narrows the geographic gap between our Canadian and Mongolian populations. The published photographic maps of the Yukon population [23] are small and incomplete, but suggest that *IS-17*,*18*,*19*; *IL-18*; *IIS-1*,*5*,*6*; *IIIS-1*; and *IIIL-2*,*4*,*23* are present. The majority of IIL is missing and the breakpoints of IIS-1 [23], which is not the same inversion as our *IIS-1*, cannot be determined. What is clear is that sex determination in the Yukon population is based on IIS, the same arm as in *S*. *nigricoxum*. Nonetheless, the Yukon Y differs by one inversion from the X, rather than two, as in *S*. *nigricoxum*, and it appears to be an inversion not seen in our material. The taxonomic status of the Yukon population vis à vis *S*. *nigricoxum* is clouded by allopatry and by (presumably) only a Y-chromosome difference; neither—alone or in combination—allows a direct test of reproductive isolation. Nonetheless, the similarities (i.e., presence of *IL-18*, similar centromere bands, and sex determination based on IIS) lead us to include the Yukon population under *S*. *nigricoxum* as an example of a Y-chromosome polymorphism.

Given the consistent chromosomal differences and nonhomologous sex chromosomes (I vs II), we recognize *S*. *decimatum* and *S*. *nigricoxum* as distinct, diagnosable species. We, therefore, revalidate the name *S*. *nigricoxum* for Nearctic populations previously recognized as *S*. *decimatum* [20] and corresponding with *S*. *nigricoxum* in the expanded sense (i.e., including the Yukon population). The type locality of *S*. *nigricoxum* is Nunavut, Coronation Gulf, Hood River, about 450 km northwest of our Site 3 and more than 1300 km east of the Yukon population.

### Significance of Interchanges

#### Taxon radiations

Natural experiments involving *de novo* interchanges have been documented in one or a few nuclei in silk glands of individual simuliid larvae of non-translocation species, although no whole-arm interchanges have been observed in these mosaic glands (i.e., glands containing typical nuclei and interchange nuclei), suggesting that they are among the rarest translocational events [19]. Yet, persistent whole-arm interchanges among extant simuliids were established independently at least 11 times. The most common (38.5%) restructured combination of the six possible types involves IS + IIL, IL + IIS (Table 3). The *S*. *cholodkovskii* lineage represents the only example of the IS + IIIL, IL + IIIS combination.

**Table 3:** Whole-arm chromosomal interchanges in the Simuliidae.

Taxonomic level	Taxon	Interchanged arms	Reference
subgenus	*Helodon*	IIL + IIIS, IIS + IIIL	[15]
species	*Helodon vernalis*	IS + IIL, IL + IIS	[15,33]
species	*Prosimulium transbrachium*	IS + IIL, IL + IIS	[33]
species group (in part)	*Prosimulium magnum* group (eastern spp.)	IS + IIS, IL + IIL	[34]
genus (in part)	*Twinnia* (western spp.)	IIL + IIIL, IIS + IIIS	[35]
species (in part)^1^	*Cnephia pallipes*	IS + II (Ce IIS+IIL), IL + II (Ce IIIS+IIIL)	[18]
genus	*Metacnephia*	IS + IIL, IL + IIS	[36]
genus^2^	*Paraustrosimulium anthracinum* (Bigot)	IIS + IIIS, IIL + IIIL	[37]
species^3^	*Simulium* (*Metomphalus*) *vorax*	IS + IIL, IL + IIS	[15]
species^4^	*Simulium* (*Pternaspatha*) *limay*	IIS + IIIS, IIL + IIIL	[38]
species subgroup	*Simulium* (*Simulium*) *cholodkovskii* lineage	IS + IIIL, IL + IIIS	[23], present study
subgenus	*Simulium* (*Wilhelmia*)	IS + IIL, IL + IIS	[15]

^1^ Males, but not females, of this dichromosomic (*n* = 2) species, formerly known as *Cnephia lapponica*, are whole-arm interchange heterozygotes in only part of their geographic range [18].

^2^ The genus is monotypic.

^3^ The interchange might be found in other members of the subgenus *Metomphalus* and possibly is homologous with that of the subgenus *Wilhelmia*, which would suggest common ancestry [15].

^4^ Whether the interchange is found in other members of the subgenus *Pternaspatha* is not known.

Whole-arm interchanges are disproportionately common in the older simuliid lineages, that is, the Prosimuliinae and other non-*Simulium* taxa. About 81% of all known extant species of simuliids are in the genus *Simulium* [39], but no more than 33% of the independent whole-arm interchanges are in this genus. Perhaps the older lineages have had more time for interchanges to occur and become established, although nearly all non-*Simulium* taxa are univoltine, whereas the majority of *Simulium* species, including all interchange examples in the genus, are capable of multiple generations annually. The non-*Simulium* taxa are predominantly cool-water inhabitants, possibly enhancing the persistence of trial mutations involving translocations.

Whole-arm interchanges in the Simuliidae are often associated with taxon radiations. The number of whole-arm interchanges shared by two or more species (6 examples) is equal to the number in singletons. The 11 or 12 independently derived examples have produced at least 59 nominal species, about 2.7% of the world’s species, with less than 25% of the total species adequately surveyed [12]. If all members of the genus *Metacnephia* and subgenera *Helodon* and *Wilhelmia* are interchange species, the percentage jumps to about 6% and progressively increases if whole-arm interchanges characterize poorly surveyed higher taxa (e.g., subgenera *Metomphalus* and *Pternaspatha*) in which interchanges have been reported from single species. Thus, once established, an interchange in an ancestral species had at least a 50% probability of being involved in subsequent speciation, based on extant examples.

An ancestor of the *S*. *cholodkovskii* lineage with the IS + IIIL, IL + IIIS interchange gave rise to a set of species that colonized a vast expanse of the northern Holarctic Region, and now often dominate the macroinvertebrate fauna of large rivers and cause pest problems for humans and livestock [30]. The ability to colonize the planet’s large rivers evolved before the origin of the interchange, having arisen in an ancestor of a much larger clade [28]. Diversification within the *S*. *cholodkovskii* lineage, perhaps facilitated by the ancestral interchange, would have allowed the lineage to capitalize further on the large-river niche. This radiation is consistent with the general observations for the origins of pest status in black flies, *viz*., colonization of large continental rivers [28,40].

### Did the interchange precipitate diversification?

In the karyotypic restructuring of the *S*. *cholodkovskii* lineage, we favor the hypothesis that the translocation event occurred early in male spermatogonia, such that the translocated chromosome complement could have been transmitted to both the genetic X- and Y-bearing sperm during spermatogenesis. Present-day differentiated sex chromosomes that demark the IIS arm of *S*. *nigricoxum* might be informative by suggesting that the IIS arm was involved in ancestral sex determination. Such a scenario in the ancestral taxon would ensure equal production of female- and male-bearing sperm, assuming random segregation of chromosome II. Alternate disjunction of translocation heterozygotes into viable gametes and subsequent mating would produce heterozygous F1 females and males and eventually F2 translocation homozygotes. These homozygotes are postulated to have had a selective reproductive advantage that eventually resulted in population expansion. Mechanistically, the breakpoints could have been proximal or distal to the centromeres of I and III. Depending on the double-strand breakpoints, rejoining of new arm configurations potentially would produce modifiers of linkage interaction (heterochromatin–euchromatin junction) both in cis and trans configurations, and potentially could change centromeric three-dimensional structural interactions (architecture), all of which could impact genic expression levels.

Our analyses and interpretation represent the first documented case in the Simuliidae in which complex and compound structural genomic rearrangements can be associated with, and factor into, the speciation process. Speciation in the *S*. *cholodkovskii* lineage encompassed three chromosomal restructuring phenomena, the most conspicuous of which was a lineage-defining reciprocal translocation event. Whole-arm interchanges have been implicated as precipitators of reproductive isolation, largely postzygotic. A case has been made that the whole-arm interchange characterizing *Prosimulium transbrachium* Adler & Kim—for which the species is named—was important in initiating its reproductive isolation [33]. Any translocations that do not segregate efficiently at meiosis can act as postmating isolating mechanisms through the production of unbalanced zygotes or infertile hybrids [41].

A second rearrangement phenomenon involved differential expression of one and the same rearrangement in different species. Phylogenetic inference indicates that IIS-4 was polymorphic in the original translocation population and was sorted out in two configurations—fixed and X linked—among extant taxa. The evolutionary acquisition of different roles of an ancestral polymorphism in derivative lineages is one of the most recurrent themes in chromosomal restructuring of species in the family Simuliidae [18,27,42,43].

The third phenomenon involved taxon-specific differentiation of sex chromosomes in the members of the *S*. *cholodkovskii* lineage. A model of sex-chromosome differentiation has been developed for the family Simuliidae, which asserts that pairs of coadapted X and Y sequences promote sympatric speciation by disruptive selection with assortative mating [11]. The three restructuring events are hypothesized to have occurred early in the differentiation and speciation process of the *S*. *cholodkovskii* lineage, as evidenced by the existence of at least three extant species and their geographic expansion over an enormous extent of the northern Nearctic and Palearctic Regions.

### Structural and Functional Interactions—Future Genomic Research

Given the wealth of chromosomal variation in the Simuliidae, and particularly the whole-arm interchange rearrangements that act as phyletic markers, the functional genomic analysis of simuliids should prove informative for extending our understanding of insect cell dynamics in relation to evolution and speciation. For the *S*. *cholodkovskii* lineage, part of such an analysis would require resolution of translocation breakpoints in the centromeric regions. Although inferences about the breakpoints might be possible at a macro-scale by C banding, a molecular approach more likely would be needed. This approach would require sequencing of centric regions and adjoining euchromatic regions, proximal and distal to the centric heterochromatin, and the use of fluorescence *in situ* hybridization (FISH) for potential physical-mapping validation with specific probes determined from the sequencing information.

The quest for blood to mature the eggs of black flies drives the economic importance of many species of the Simuliidae, including members of the *S*. *cholodkovskii* lineage, which are among the most virulent pests in the entire family [28]. Thus, comparative genomic research could be undertaken on the sialomes of adult simuliids [44,45], using potentially informative taxonomic groups, such as the *S*. *cholodkovskii* lineage, to further elucidate the evolution of blood feeding in black flies. This research approach should be feasible, given that functional genes and their protein products in the sialome appear to be under various degrees of selection, as inferred by species differences in biological characteristics, such as autogeny, biting behavior (i.e., ornithophily and zoophily including anthropophily), vectorial capacity, and biochemical activity of factors in the coagulation pathway [46], and because of their suitability as informative heterologous cDNA probes. Applications also are possible for the interdiction of simuliid vector sialomes of human onchocerciasis and their products during parasite transmission [17,45]; similar possibilities also hold for other vector-group sialomes, such as those of mosquitoes [47].

Sequencing of the genome, identification and characterization of genes, physical mapping of genes to determine linkage associations, and the relationship between microchanges (e.g., indels) versus macro changes, such as the inversions and translocations observed in our study, would be needed before studies on the epigenome could be undertaken to investigate cellular structure-function relationships. Similar translocation phenomena in mosquitoes, associated with progress toward using a high-resolution chromosome map in physical genome mapping, provide a direction forward for simuliid genomic studies [48,49].

In this regard, some information is available on basic nuclear ultrastructure of interphase and meiotic divisions in the Simuliidae. Chromosome organization generally follows a Rabl orientation during mitosis and a bouquet arrangement during meiosis, with the telomeres of homologous paired chromosomes attaching to the nuclear membrane and the centromeres being centrally located in the nucleus. In the Rabl orientation, telomeric attachments of homologues are spaced farther away, whereas in the bouquet arrangement, the telomeres of metacentric bivalents loop back on themselves such that the telomeric ends of each bivalent are attached to the nuclear envelope in close apposition [50]. Like achiasmate males of *D*. *melanogaster* [51,52], males of the black fly *Cnephia dacotensis* (Dyar & Shannon) lack synaptonemal complexes during zygotene/pachytene, exhibit no chiasmata, and lack genetic recombination [53]. On the other hand, females in related dipteran families form synaptonemal complexes during meiotic prophase, are chiasmate, and exhibit genetic recombination. In *D*. *melanogaster*, the bivalents remain Rabl like in orientation, but in *C*. *dacotensis*, bivalent orientation is equivocal. A complete three-dimensional reconstruction of each bivalent was not feasible for *C*. *dacotensis* even though telomeric ends of bivalents attached to the nuclear envelope, with the centric region of one partially traceable metacentric bivalent being centrally located in the nucleus [53].

## References

[1] Li H, Fan R, Fu S, Wei B, Xu S, Feng J, et al (2015) Development of *Triticum aestivum*–*Leymus mollis* translocation lines and identification of resistance to stripe rust. Journal of Genetics and Genomics 42: 129–132. 10.1016/j.jgg.2014.11.008 25819090

[2] Türkösi E, Farkas A, Aranyi NR, Hoffmann B, Tóth V, Molnár-Láng M (2015 [2014]) Improvement of the agronomic traits of a wheat-barley centric fusion by introgressing the 3HS.3BL translocation into a modern wheat cultivar. Genome 57: 601–607. 10.1139/gen-2014-0187 25806585

[3] Roukos V, Voss TC, Schmidt CK, Lee S, Wangsa D, Misteli T (2013) Spatial dynamics of chromosome translocations in living cells. Science 341: 660–664. 10.1126/science.1237150 23929981 PMC6324928

[4] Schwartz M, Hakim O (2014) 3D view of chromosomes, DNA damage, and translocations. Current Opinion in Genetics and Development 25:118–125. 10.1016/j.gde.2013.12.008 24632298

[5] Golczyk H, Massouh A, Greiner S (2014) Translocations of chromosome end-segments and facultative heterochromatin promote meiotic ring formation in evening primroses. Plant Cell 26: 1280–1293. 10.1105/tpc.114.122655 24681616 PMC4001384

[6] Garagna S, Page J, Fernandez-Donoso R, Zuccotti M, Searle JB (2014) The Robertsonian phenomenon in the house mouse: mutation, meiosis and speciation. Chromosoma 123: 529–544. 10.1007/s00412-014-0477-6 25053180

[7] Stephens PJ, Greenman CD, Fu B, Yang F, Bignell GR, Mudie LJ, et al (2011) Massive genomic rearrangement acquired in a single catastrophic event during cancer development. Cell 144: 27–40. 10.1016/j.cell.2010.11.055 21215367 PMC3065307

[8] Wang J, Lan X, Hsu P-Y, Hsu H-K, Huang K, Parvin J, et al (2013) Genome-wide analysis uncovers high frequency, strong differential chromosomal interactions and their associated epigenetic patterns in E2-mediated gene regulation. BMC Genomics 14: 70 10.1186/1471-2164-14-70 23368971 PMC3599885

[9] Harewood L, Fraser P (2014) The impact of chromosomal rearrangements on regulation of gene expression. Human Molecular Genetics 23 (R1): R76–R82. 10.1093/hmg/ddu278 24907073

[10] Bohlander SK, Kakadia PM (2015) DNA repair and chromosomal translocations In Ghadimi BM, Ried T, editors. Chromosomal instability in cancer cells. Recent Results in Cancer Research 200: 1–37. 10.1007/978-3-319-20291-4_1 26376870

[11] Rothfels K (1989) Speciation in black flies. Genome 32: 500–509.

[12] Adler PH, Crosskey RW (2015) Cytotaxonomy of the Simuliidae (Diptera): a systematic and bibliographic conspectus. Zootaxa 3975: 1–139. 10.11646/zootaxa.3975.1.1 26249931

[13] Conflitti IM, Shields GF, Murphy RW, Currie DC (2015) The speciation continuum: ecological and chromosomal divergence in the *Simulium arcticum* complex (Diptera: Simuliidae). Biological Journal of the Linnean Society 115: 13–27. 10.1111/bij.12480

[14] Bedo DG (1977) Cytogenetics and evolution of *Simulium ornatipes* Skuse (Diptera: Simuliidae). I. Sibling speciation. Chromosoma 64: 37–65.

[15] Rothfels KH (1979) Cytotaxonomy of black flies (Simuliidae). Annual Review of Entomology 24: 507–539.

[16] Procunier WS, Post RJ (1986) Development of a method for the cytological identification of man-biting sibling species within the *Simulium damnosum* complex. Tropical Medicine and Parasitology 37: 49–53. 3704475

[17] Procunier W, Zhang D, Cupp MS, Miller M, Cupp EW (2005) Chromosomal localization of two antihemostatic salivary factors in *Simulium vittatum* (Diptera: Simuliidae). Journal of Medical Entomology 42: 805–811. 16363163 10.1093/jmedent/42.5.805

[18] Procunier WS (1982a) A cytological study of species in *Cnephia* s. str. (Diptera: Simuliidae). Canadian Journal of Zoology 60: 2866–2878. 10.1139/z75-196 1192297

[19] Rothfels KH (1980) Chromosomal variability and speciation in blackflies *In* Blackman RL, Hewitt GM, Ashburner M, editors. Insect cytogenetics. Symposium of the Royal Entomological Society of London, Number 10. Oxford: Blackwell Scientific Publications, pp. 207–224.

[20] Adler PH, Currie DC, Wood DM (2004) The black flies (Simuliidae) of North America Ithaca, NY: Cornell University Press.

[21] Procunier WS, Muro AI (1994) A mid-arm interchange as a potential reproductive isolating mechanism in the medically important *Simulium neavei* group (Diptera: Simuliidae). Genome 37: 957–969. 7828843 10.1139/g94-136

[22] Bedo DG (1975) Polytene chromosomes of three species of blackflies in the *Simulium pictipes* group (Diptera: Simuliidae). Canadian Journal of Zoology 53: 1147–1164. 1156954 10.1139/z75-134

[23] Shields GF (1990) Interchange chromosomes in *Simulium nigricoxum* Stone Diptera: Simuliidae. Genome 33: 683–685.

[24] Adler PH, Huang YT (2011) Integrated systematics of the Simuliidae (Diptera): evolutionary relationships of the little-known Palearctic black fly *Simulium acrotrichum*. Canadian Entomologist 143: 612–628. 10.4039/n11-035

[25] Rubtsov IA (1956) Moshki (sem. Simuliidae) [Blackflies (fam. Simuliidae)]. Fauna of the USSR. New Series No. 64, Insects, Diptera 6 (6). Moscow & Leningrad [= St. Petersburg]: Akademii Nauk SSSR. [In Russian; English translation: 1990. Blackflies (Simuliidae). 2^nd^ ed. Fauna of the USSR. Diptera, 6 (6). Leiden: E. J. Brill].

[26] Charalambous M, Shelley AJ, Maia Herzog M, Luna Dias APA (1996) Four new cytotypes of the onchocerciasis vector blackfly *Simulium guianense* in Brazil. Medical and Veterinary Entomology 10: 111–120. 8744702 10.1111/j.1365-2915.1996.tb00716.x

[27] Rothfels K, Feraday R, Kaneps A (1978) A cytological description of sibling species of *Simulium venustum* and *S*. *verecundum* with standard maps for the subgenus *Simulium* Davies [*sic*] (Diptera). Canadian Journal of Zoology 56: 1110–1128.

[28] Adler PH, Kúdelová T, Kúdela M, Seitz G, Ignjatović-Ćupina A (2016) Cryptic biodiversity and the origins of pest status revealed in the macrogenome of *Simulium colombaschense* (Diptera: Simuliidae), history’s most destructive black fly. PLoS ONE 11(1): 1–25 e0147673. 10.1371/journal.pone.0147673 PMC4726606 26808274

[29] Yankovsky AV (1995) Family Simuliidae Newman. In: Yankovsky AV, Ulyanov KN, compilers. Catalogue of type specimens in the collection of the Zoological Institute, Russian Academy of Sciences [Katalog tipovykh ekzemplyarov kollektsii Zoologicheckogo Instituta RAN. Diptera. 5. Simuliidae, Culicidae. St. Petersburg, Russia: Rossiiskaya Akademiya Nauk, Zoologicheskii Institut, pp. 1–61. In Russian.

[30] Halgoš J, Jedlička L, Cendsuren A (1982) Black flies (Diptera, Simuliidae) in the Selenge River Basin on the territory of Mongolian People’s Republic: in memory of Professor Anuudarijn Dasdorz. Acta Facultatis Rerum Naturalium Universitatis Comenianae—Zoologia 26: 1–52.

[31] Rothfels KH, Featherston D (1981) The population structure of *Simulium vittatum* (Zett.): the IIIL-1 and IS-7 sibling species. Canadian Journal of Zoology 59: 1857–1883.

[32] Adler PH, Cheke RA, Post RJ (2010) Evolution, epidemiology, and population genetics of black flies (Diptera: Simuliidae). Infection, Genetics and Evolution 10: 846–865. 10.1016/j.meegid.2010.07.003 20624485

[33] Rothfels K, Freeman DM (1983) A new species of *Prosimulium* (Diptera: Simuliidae): an interchange as a primary reproductive isolating mechanism? Canadian Journal of Zoology 61: 2612–2617.

[34] Ottonen PO, Nambiar R (1969) The salivary gland chromosomes of *Prosimulium multidentatum* Twinn and three forms included in *Prosimulium magnum* (Dyar and Shannon) (Diptera: Simuliidae). Canadian Journal of Zoology 47: 943–949.

[35] Rothfels K, Freeman M (1966) The salivary gland chromosomes of three North American species of *Twinnia* (Diptera: Simuliidae). Canadian Journal of Zoology 44: 937–945. 5919300 10.1139/z66-095

[36] Procunier WS (1982b) A cytological description of 10 taxa in *Metacnephia* (Diptera: Simuliidae). Canadian Journal of Zoology 60: 2852–2865.

[37] Rothfels KH (1988 [1987]) Cytological approaches to black fly taxonomy In: Kim KC, Merritt RW, editors. Black flies: ecology, population management, and annotated world list. University Park, PA: Pennsylvania State University Press, pp. 39–52.

[38] Coscarón-Arias CL (2000) The salivary gland chromosomes of the black fly *Simulium* (*Pternaspatha*) *limay* (Diptera: Simuliidae) from Argentina. Revista Colombiana de Entomología 26: 137–143.

[39] Adler PH, Crosskey RW (2016) World blackflies (Diptera: Simuliidae): a comprehensive revision of the taxonomic and geographical inventory [2016]. 126 pp. Available: http://www.clemson.edu/cafls/biomia/pdfs/blackflyinventory.pdf Accessed 6 May 2016.

[40] Shields GF, Hokit DG (2016) Does river corridor affect chromosome forms within the black fly *Simulium arcticum* complex (Diptera: Simuliidae)? Freshwater Science 35: In press. 10.1086/686913

[41] King M (1993) Species evolution: the role of chromosome change Cambridge: Cambridge University Press.

[42] Tangkawanit U, Kuvangkadilok C, Baimai V, Adler PH (2009) Cytosystematics of the *Simulium tuberosum* group (Diptera: Simuliidae) in Thailand. Zoological Journal of the Linnean Society 155: 289–315. 10.1111/j.1096-3642.2008.00433.x

[43] Adler PH, Cherairia M, Arigue SF, Samraoui B, Belqat B (2015) Cryptic biodiversity in the cytogenome of bird-biting black flies in North Africa. Medical and Veterinary Entomology 29: 276–289. 10.1111/mve.12115 25801314

[44] Ribeiro JMC, Valenzuela JG, Pham VM, Kleeman L, Barbian KD, Favreau AJ, et al (2010) An insight into the sialotranscriptome of *Simulium nigrimanum*, a black fly associated with fogo selvagem in South America. American Journal of Tropical Medicine and Hygiene 82: 1060–1075. 10.4269/ajtmh.2010.09-0769 20519601 PMC2877412

[45] Chagas AC, Calvo E, Pimenta PF, Ribeiro JM (2011) An insight into the sialome of *Simulium guianense* (Diptera: Simuliidae), the main vector of River Blindness Disease in Brazil. BMC Genomics 12: 612 10.1186/1471-2164-12-612 22182526 PMC3285218

[46] Cupp MS, Ribeiro JMC, Cupp EW (1994) Vasodilative activity in black fly salivary glands. American Journal of Tropical Medicine and Hygiene 50: 241–246. 8116819 10.4269/ajtmh.1994.50.241

[47] Sharakhov IV, Sharakhova MV (2015) Heterochromatin, histone modifications, and nuclear architecture in disease vectors. Current Opinion in Insect Science 10: 110–117. 10.1016/j.cois.2015.05.003 26097808 PMC4470418

[48] Sharakhova MV, Peery A, Antonio-Nkondjio C, Xia A, Ndo C, Awono-Ambene P, et al (2013) Cytogenetic analysis of *Anopheles ovengensis* revealed high structural divergence of chromosomes in the *Anopheles nili* group. Infection, Genetics and Evolution 16: 341–348. 10.1016/j.meegid.2013.03.010 23523820 PMC3669242

[49] Artemov GN, Sharakhova MV, Naumenko AN, Karagodin DA, Baricheva EM, Stegniy VN, Sharakhov IV (2015) A standard photomap of ovarian nurse cell chromosomes in the European malaria vector *Anopheles atroparvus*. Medical and Veterinary Entomology 29: 230–237. 10.1111/mve.12113 25776224 PMC4515173

[50] Cowan CA, Carlton PM, Cande WZ (2001) The polar arrangement of telomeres in interphase and meiosis—Rabl organization and the bouquet. Plant Physiology 125: 532–538. 11161011 10.1104/pp.125.2.532 PMC1539364

[51] Obeso D, Pezza RJ, Dawson D (2014) Couples, pairs, and clusters: mechanisms and implications of centromere associations in meiosis. Chromosoma 123: 43–55. 10.1007/s00412-013-0439-4 24126501 PMC3969401

[52] Vazquez J, Belmont AS, Sedat JW (2002) The dynamics of homologous chromosome pairing during male *Drosophila* meiosis. Current Biology 12: 1473–1483. 10.1016/S0960-9822(02)01090-4 12225662

[53] Procunier WS (1975) A cytological study of two closely related blackfly species: *Cnephia dacotensis* and *Cnephia ornithophilia* (Diptera: Simuliidae). Canadian Journal of Zoology 53: 1622–1637. 1192297 10.1139/z75-196

